# Seasonal dynamics of water use between *Eucalyptus globulus* and *Pinus massoniana* plantations in karst graben basins in China

**DOI:** 10.3389/fpls.2026.1864566

**Published:** 2026-07-08

**Authors:** Zihan Xu, Ming Cui, Sinsin Brice, Ziqing Zhao, Jiahao Li, Shuangxi Zhou, Xinjun Chen, Shujun Cui, Yuguo Liu

**Affiliations:** 1 Xichuan Research Station, Institute of Ecological Conservation and Restoration, Chinese Academy of Forestry, Beijing, China; 2 Natural Resource Conservation, University of Abomey-Calavi, Cotonou, Benin; 3 Research Institute of Subtropical Forestry, Chinese Academy of Forestry, Hangzhou, China; 4 Department of Biological Sciences, Macquarie University, Sydney, NSW, Australia; 5 Forestry and Grassland Bureau of Weichang Manchu and Mongolian Autonomous County, Chengde, China

**Keywords:** climate resilience, drought, ecosystem restoration, karst, MixSIAR model, soil water stress, stable isotopes, water use efficiency

## Abstract

Karst regions are among the area most severely affected by rocky desertification and are recognized as some of the world’s most ecologically fragile landscapes. Graben basins represent a distinctive geomorphic unit in the karst region of Southwest China, where large-scale afforestation faces a fundamental conflict between plant water demand and limited soil-water availability. However, the seasonal dynamics of transpiration and water uptake patterns of plantation species in these drought-prone landscapes remain poorly quantified. In this study, we investigated two representative plantation species, *Eucalyptus globulus* and *Pinus massoniana*, in a karst graben basin of Southwest China. By integrating sap flow monitoring with stable isotope analysis, we quantified seasonal variations in plant water sources and transpiration. The results revealed pronounced seasonal and species-level differences in water use between the two plantations. During the dry season, *E. globulus* primarily obtained water from the 20–100 cm soil layer, which accounted for 68.2% of its total water uptake, indicating a relatively flexible water use strategy. In contrast, *P. massoniana* mainly relied on the 0–60 cm soil layers, which contributed 70.4% of its water uptake, suggesting a more conservative strategy. During the rainy season, with increased precipitation input, both plantations preferentially used shallow soil water from the 0–20 cm layer. Transpiration was most active during the rainy season. Monthly mean transpiration of *E. globulus* was 2.1 and 1.5 times that of *P. massoniana* in the dry and rainy seasons, respectively. Overall, during the observation period, both plantation types adjusted their water uptake depth and transpiration processes in response to seasonal changes in water availability. These findings provide evidence for understanding soil-plant water relations in plantation ecosystems of karst graben basins and offer practical insights for site-specific species selection and plantation water management. Nevertheless, the long-term ecological and hydrological effects of these plantations require further evaluation through multi-year monitoring.

## 1 Introduction

The karst region of Southwest China is governed by a coupled surface-subsurface hydrological system and is generally characterized by shallow and discontinuous soil layers, rapid water leakage, deeply buried groundwater, and strong spatial heterogeneity in soil and water resources. These features jointly constrain vegetation recovery and ecosystem stability (Yuan, 1997; Jiang et al., 2014; Peng and Ding, 2023; Jiang et al., 2024; Zhang et al., 2024). Within this region, karst graben basins represent a distinctive geomorphic unit, occurring mainly in eastern Yunnan, the Panxi region of southwestern Sichuan, and western Guizhou (Li et al., 2020). Unlike typical karst mountain landscapes, where soils are thin and bedrock is widely exposed, graben basins are characterized by sharp topographic contrasts between basin floors and surrounding mountains, strong spatial heterogeneity in soil and water resources, and deep groundwater tables (Cao, 2021; Wang et al., 2017; Zhang et al., 2025). Basin floors often contain relatively deep soils formed by long-term hillslope erosion and deposition. However, deep soils do not necessarily provide a sustained and reliable water supply. Precipitation can rapidly bypass the root zone through carbonate fissures, conduits, and epikarst pathways, thereby shortening soil water residence time and weakening the sustained water supply available to plants (Dai et al., 2018; Al Khoury et al., 2023). Consequently, plants growing in karst graben basins may face a fundamental paradox: relatively deep rooting space coexists with chronically limited effective water availability.

To mitigate rocky desertification and promote the recovery of degraded karst ecosystems, afforestation has been widely implemented across this region. Among the species used, fast-growing plantation trees have been widely adopted because they can rapidly increase vegetation cover and improve surface environmental conditions in the short term. However, plantation restoration may also place considerable pressure on regional water resources, particularly in karst landscapes where precipitation is the primary water input and subsurface leakage is pronounced. If plantation establishment exceeds the regional water-carrying capacity, high transpiration demand may intensify soil water depletion and increase the risk of stand decline (Pei et al., 2023a). *Eucalyptus globulus* and *Pinus massoniana* are two representative pioneer species used for afforestation in karst graben basins. *E. globulus* is characterized by rapid growth, strong adaptability, and high transpiration potential, whereas *P. massoniana* has long been used in ecological restoration because of its strong environmental tolerance (Shu et al., 2004; Huang et al., 2013). Therefore, comparing these two plantation types can help reveal how different tree species regulate water uptake and transpiration under the same hydrological constraints.

Stable isotope tracing has become an important approach for identifying plant water sources by comparing the hydrogen and oxygen isotope compositions of xylem water with those of potential source waters (Dawson et al., 2002; Sprenger et al., 2016). Previous studies in karst ecosystems have shown that plants generally rely mainly on shallow soil water during the rainy season but shift toward deeper soil water, fissure water, or epikarst water during dry season, indicating strong seasonal plasticity in water uptake (Liu et al., 2014; Nie et al., 2014; Deng et al., 2015). However, most existing studies have focused on conventional karst landscapes, such as peak cluster depressions, whereas plant water use under the distinctive geomorphic and hydrological setting of karst graben basins remains poorly understood. To date, research on plant water use in these basins has mainly been limited to soil water uptake by apple trees at different growth stages (Huo, 2020). For the plantation forests widely established in this region, quantitative assessments of water source partitioning and actual transpiration consumption are still lacking. This knowledge gap limits our ability to evaluate how different plantations respond to seasonal drought and how they may affect regional water resources.

Based on this context, this study investigated *E. globulus* and *P. massoniana* plantations in a karst graben basin of Southwest China. By integrating stable hydrogen and oxygen isotope analysis, the MixSIAR mixing model, and sap flow monitoring, we systematically evaluated the seasonal water sources and transpiration consumption of the two plantation types. The specific objectives were to: (1) quantify the relative contributions of soil water from different depths to water uptake by the two plantation species during the dry and rainy seasons; (2) compare stand-level transpiration between the two plantation types based on sap flow measurements; and (3) clarify the implications of contrasting water-use strategies for plantation management and species selection in karst graben basins.

## 2 Materials and methods

### 2.1 Study area

**Figure 1:**
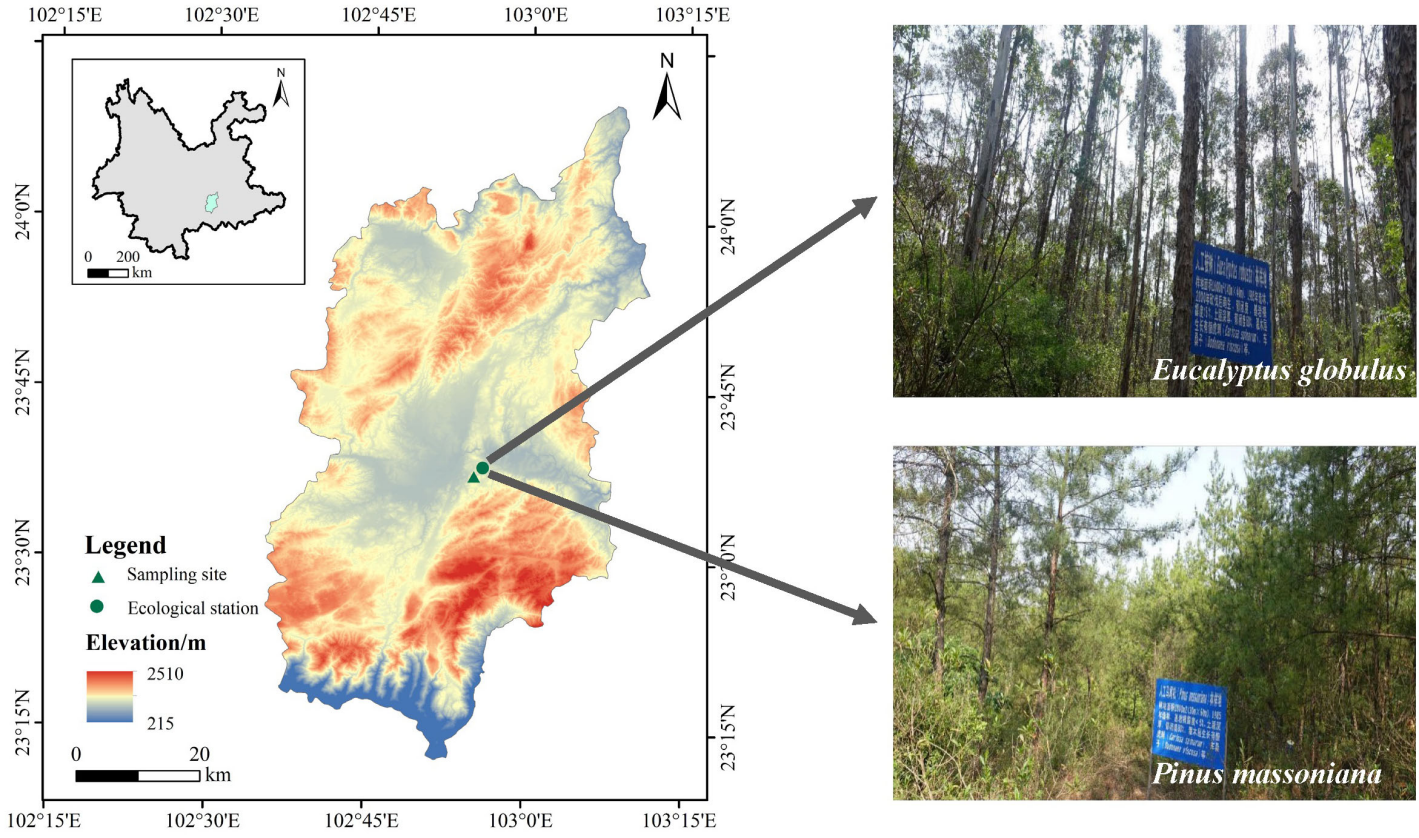
Overview map of the research area.

The study area is located in the Jianshui County, southern Yunnan Province, China, within the Jiubiao Experimental Forest Farm of the Yunnan Jianshui Desert Ecosystem National Observation and Research Station (23°36′50″–23°37′30″ N, 102°54′00″–102°54′55″ E) (**Figure 1**). This area is characterized by a typical karst graben basin landform, with elevations ranging from 1,350 to 1,700 m. The region has a subtropical monsoon climate influenced by the southwest Indian Ocean monsoon, with distinct dry and rainy seasons. The highly uneven distribution of precipitation results in pronounced seasonal drought. Mean annual precipitation is 827.6 mm (1991-2020), with rainfall concentrated mainly during the rainy season from June to September. The mean annual temperature is 19.8 °C, mean annual evaporation reaches 1,800 mm, and mean annual relative humidity of 68%. The region shows a marked imbalance between water and heat, as evaporative demand far exceeds precipitation supply. The parent materials are mainly limestone-derived calcareous soils and Quaternary red earths, and the basin dominated by relatively deep red alluvial soils. Afforestation at the forest farm began in 2000. *Eucalyptus globulus* and *Pinus massoniana*, both evergreen tree species, are widely used for afforestation in Southwest China. The *E. globulus* plantation plot covers an area of 40 m × 40 m (102°54′47″ E, 23°37′19″ N), and the *P. massoniana* plantation plot covers an area of 30 m × 60 m (102°54′23″ E, 23°37′11″ N). Dominant understory species include *Dodonaea viscosa*, *Osteomeles anthyllidifolia*, and *Lantana camara*. Basic information on the sample plots is provided in **Table 1**.

**Table 1:** Basic information of sample plots.

Plot	Slope (°)	Aspect	Average crown width (m)	Average tree height (m)	Average DBH (cm)	Average sapwood area (cm^2^)	pH
Eucalyptus globulus	2	north by east	2.7 ± 0.8	14.2 ± 4.2	13.8 ± 6.4	198.9 ± 59.8	5.9
Pinus massoniana	2	north	1.6 ± 0.5	11.5 ± 3.8	12.9 ± 6.0	117.7 ± 24.0	5.4

### 2.2 Experimental design and sample collection

The study area is situated on the floor of a karst graben basin, where slope erosion and deposition have produced relatively deep soils (Zhang et al., 2007; Ji et al., 2023; Zhang et al., 2025). The 1 m soil profile contained no continuous bedrock, coarse fragments, or visible fissures. A groundwater table deeper than 60 m inferred from domestic well depths lies beyond root access. Soil water within the 0-100 cm profile was therefore considered the principal potential water source for the two plantation species.

Soil and xylem samples were collected monthly in the middle of each month from May to October 2021. For xylem sampling, three representative trees were selected from each stand based on mean stand height and diameter at breast height (DBH). Healthy branches were collected from each tree in the morning between 08:00 and 11:00. Stem segments at least 5 cm away from foliage-bearing nodes were cut into 3–5 cm lengths, and the phloem was immediately removed. The xylem samples were placed in 50 mL centrifuge tubes and stored for stable isotope analysis. A total of 36 xylem samples were collected during the study period.

Soil samples were collected simultaneously with xylem samples using a manual soil auger with a diameter of 5 cm. Soil cores were collected approximately 0.5 m from the stem base of each sampled tree at six depth intervals: 0-10, 10-20, 20-40, 40-60, 60-80, and 80–100 cm. Three replicates were collected from each soil layer. All soil samples were placed in 50 mL centrifuge tubes, frozen, and stored for stable isotope analysis. A total of 180 soil samples were collected.

Atmospheric precipitation samples for isotope analysis were collected using a funnel device placed in an open area approximately 100 m from the study plots. Rainwater samples were collected immediately after each precipitation event >1 mm and sealed in 50 mL centrifuge tubes for subsequent analysis. A total of 29 rainwater samples were collected.

### 2.3 Isotope sample measurement and analysis

Water was extracted from plant xylem and soil samples using a vacuum condensation extraction system (Li-2100, LICA, China). The δD and δ¹^8^O values of the extracted water were measured with a liquid water isotope analyzer (DLT-100, LGR, USA), with analytical precisions of ± 0.1‰ for δ¹^8^O and ± 0.3‰ for δD. The isotope ratios are reported in δ notation (‰) relative to vienna standard mean ocean water (V-SMOW) (Coplen, 2011), calculated using the following formula:


$$\delta_{sample}=\frac{(R_{sample}-R_{standard})\times 1000}{R_{standard}}$$

In the equation, $\delta_{sample}$ represents the measured δD and δ^18^O values; R_sample_ and R_standard_ represent the ^2^H/H and ^18^O/^16^O ratios of the sample and standard, respectively.

The magnitude of the line-conditioned excess (lc-excess) offset reflects the extent to which the plant’s water supply is affected by evapotranspiration, as shown by the following formula:


$$lc-excess=\delta D-(a\times \delta^{18}O+b)$$

In the equation, a and b represent the slope and intercept of the local meteoric water line (LMWL), respectively (Landwehr and Coplen, 2006).

To quantify the relative contributions of soil water from different depths to plant water uptake, we used the Bayesian mixing model MixSIAR in R v4.1.1. Xylem water δD and δ¹^8^O values were used as mixture data, and soil water from six depth intervals, 0-10, 10-20, 20-40, 40-60, 60-80, and 80–100 cm, was defined as potential source water. Isotopic analyses were conducted separately for each sampling month from May to October, using the monthly means and standard deviations of soil water isotopic values as source inputs. Soil depth was treated as the source category. Because isotopic fractionation during root water uptake was assumed to be negligible, discrimination values for both isotopes were set to zero. The model included both residual and process errors and used an uninformative Dirichlet prior, α = 1. MCMC simulations were run with three parallel chains of 300,000 iterations, and convergence was confirmed using the Gelman-Rubin diagnostic (R̂ < 1.05). To facilitate ecological interpretation, the six water sources were further grouped into three depth intervals: shallow (0–20 cm); middle (20–60 cm); and deep (60–100 cm) soil water sources (Pei et al., 2023b).

### 2.4 Measurement of sap flow in tree trunks

Six healthy trees were selected from each stand of *E. globulus* and *P. massoniana*. Sap flow was monitored using thermal dissipation probes (TDP; Plantsensor, Australia) constructed following Granier’s design. Each probe pair consisted of two 30 mm long, 2 mm diameter needles: the upper needle served as the heater and the lower needle as the unheated reference. For each tree, one probe pair was installed on the north-facing side of the stem at breast height (1.3 m), with the two probes inserted vertically into the sapwood at a spacing of 12 cm. After installation, the probes were shielded with reflective and insulating materials to prevent solar heating and rainwater intrusion. The voltage difference between the heated and reference probes was scanned every 60 s, and 10 min averages were recorded using a data logger (RR-1016, China). Because only one probe set was installed on each tree, sap flow velocity measured on the north-facing side of the trunk was assumed to represent the whole-tree average. This assumption is supported by previous studies showing that sap flow velocity measured on the northern aspect and at a sapwood depth of 2–4 cm can provide a reliable proxy for mean sap flow velocity across azimuthal directions and radial profile (Han et al., 2019; Qin et al., 2022; Liu et al., 2024). Sap flow velocity, $J_{s}$, cm·h^-^¹, was calculated using the empirical equation established by Granier (1987):


$$J_{s}=118.99\times 10^{-6}\times {\frac{\Delta T_{max}-\Delta T}{\Delta T}}^{1.231}\times 3600$$

where ΔT represents the actual temperature difference between the heated and reference probes (°C), and $\Delta T_{max}$ is the maximum temperature difference between the two probes. Under the assumption that sap flow velocity did not vary across radial depths or azimuthal orientations, individual tree water consumption was calculated using the following formula.

To estimate individual tree water consumption and stand-level transpiration, accurate determination of sapwood area at breast height was required. To avoid damaging the sampled trees, 15 healthy individuals of *E. globulus* and *P. massoniana* were selected near the sample plots. Tree cores were extracted at breast height, 1.3 m, using an increment borer, and sapwood thickness was measured with a digital caliper. Power-law relationships between diameter at breast height (DBH) and sapwood area ( $A_{s}$) were then established. For *E. globulus*, the relationship was $A_{s}$ = 0.8122 DBH^1.8533^, R² = 0.98, n = 15; for *P. massoniana*, the relationship was $A_{s}$ = 0.3971 DBH^2.067^, R² = 0.99, n = 15). Stand density was 1,450 trees ha^-^¹ in the *E. globulus* plantation and 2,527 trees ha^-^¹ in the *P. massoniana* plantation.


$$A_{s}=\pi [{\left(\frac{DBH}{2}-r_{1}\right)}^{2}-{\left(\frac{DBH}{2}-r_{2}\right)}^{2}]$$

Where $A_{s}$is the sapwood area of the sample tree (cm²), DBH is the diameter at breast height of the sample tree (cm), $r_{1}$ is the bark thickness of the sample tree (cm), $r_{2}$ is the sapwood thickness.

According to the method described by Liu et al. (2023), sap flow velocity ( $J_{s}$) was first multiplied by sapwood area ( $A_{s}$) to estimate water use by an individual sampled tree per unit time. The individual-tree water use was then scaled to the stand level using stand density ( D). Stand transpiration was calculated as follows:


$$T_{r}=\sum_{i=1}^{n=24}J_{s}\times A_{s}\times D$$

where $T_{r}$ represents the transpiration water consumption at the whole stand scale (mm), i represents the hour of the day (h), and D is the stand density (trees ha^-1^), 10–^3^ is the unit conversion coefficient.

### 2.5 Soil water content measurement

Soil moisture sensors (EC-5, METER Group, USA) were installed in both the *Eucalyptus globulus* and *Pinus massoniana* stands. A CR800 data logger (Campbell Scientific, Inc., USA) was used to continuously record soil water content at different depths. In both stands, soil moisture was measured to a depth of 60 cm and divided into four layers: 0-5, 5-20, 20-40, and 40–60 cm. Measurements were recorded every 10 min, and the daily mean values were used for subsequent analysis.

### 2.6 Root collection and analysis

In each stand, three trees of similar size were selected. A 1 m-deep soil profile was excavated 50 cm south of the trunk base of each selected tree. Undisturbed soil cores were collected from five depth intervals, 0-20, 20-40, 40-60, 60-80, and 80–100 cm, using a custom soil auger with an inner diameter of 10 cm. Roots were sieved through a 0.25 mm mesh, rinsed, and separated into fine, <2 mm; medium, 2–5 mm; and coarse, >5 mm diameter classes. The root samples were dried at 65 °C to constant mass, and dry biomass was then weighed. Because fine roots are the primary organs responsible for water and nutrient uptake, fine-root biomass in each soil layer was expressed as a percentage of total fine-root biomass within the 0–100 cm profile to characterize its vertical distribution.

### 2.7 Data analysis

The observational data used in this study covered the period from May to October 2021. A data logger malfunction in October 2021 prevented further data collection. Consequently, all analyses were based on valid measurements obtained during the May-October observation period. Seasonal transpiration was compared using one-way analysis of variance (ANOVA). Normality and homogeneity of variance were assessed using the Shapiro-Wilk test and residual diagnostics, respectively. Fine-root biomass proportion was analyzed using a linear mixed-effects model, with plantation type, soil layer, and their interaction treated as fixed effects, and individual tree identity included as a random effect. The MixSIAR analysis was performed in R v4.1.1 (R Core Team, 2021). All figures were generated using Origin 2021.

## 3 Results

### 3.1 Characteristics of atmospheric precipitation and soil moisture content

**Figure 2:**
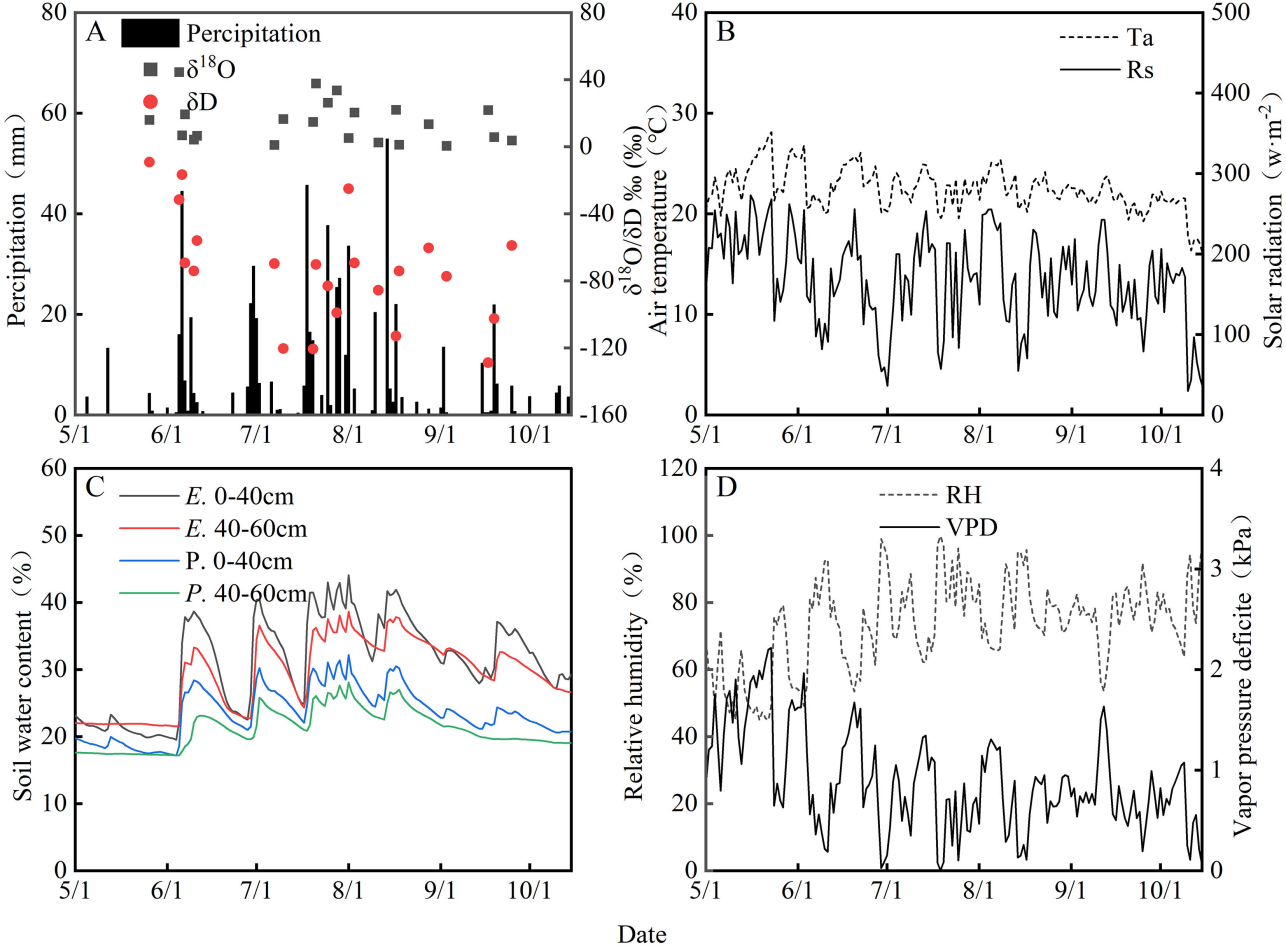
Presents the daily variations in various environmental and isotopic variables. **(A)** shows daily precipitation and the corresponding δD and δ¹^8^O values of precipitation from May to November in the study area. **(B)** illustrates the daily dynamics of air temperature (Ta) and solar radiation (Rs). **(C)** displays the volumetric water content of different soil layers in the two plantation stands, with distinct colors representing different soil depths. **(D)** depicts the daily variations in relative humidity (RH) and vapor pressure deficit (VPD). In the figure, *E.* denotes *E. globulus*, and *P.* denotes *P. massoniana*.

During the observation period from May to October 2021, monthly mean air temperature ranged from 17.83 °C to 23.91 °C, and total precipitation reached 686.2 mm. Precipitation showed a distinct seasonal pattern, with approximately 87% of total rainfall occurring from June to September, 597.0 mm. The highest monthly precipitation was recorded in July, at 225.8 mm, whereas May and October received substantially less rainfall (**Figure 2A**). Consistent with this precipitation pattern, volumetric water content (VWC) in both plantation stands was generally higher from June to September and lower in May and October. Therefore, based on the combined seasonal dynamics of precipitation and VWC during the observation period, June to September was defined as the rainy season, whereas May and October were defined as the dry season (Wang et al., 2025). The isotopic composition of precipitation varied considerably, with δD values ranging from −128.76‰ to −2.46‰, with a mean of −66.19‰, and δ¹^8^O values ranging from −18.03‰ to 1.29‰, with a mean of −9.47‰.

During the study period, soil VWC differed significantly between the upper and lower soil layers in both plantation stands (*p* < 0.05). Soil moisture in both plots fluctuated in response to precipitation events, with broadly consistent VWC dynamics between the 0–40 cm and 40–60 cm layers across the two stands. Notably, VWC in the 0–40 cm layer responded more rapidly and sensitively to rainfall than that in the 40–60 cm layer (**Figure 2C**). In the *E. globulus* stand, mean VWC was 25.18% in the dry season and 33.07% in the rainy season; in the *P. massoniana* stand, the corresponding values were 19.68% and 24.42%, respectively. Vapor pressure deficit (VPD), a key driver of stomatal regulation, transpiration, and photosynthetic, ranged from 0 to 4.8 kPa during the study period, with a mean value of 1.2 kPa, and showed a pattern opposite to that of relative humidity (RH) (**Figure 2D**).

### 3.2 Seasonal variations in water isotopes from different sources

**Figure 3:**
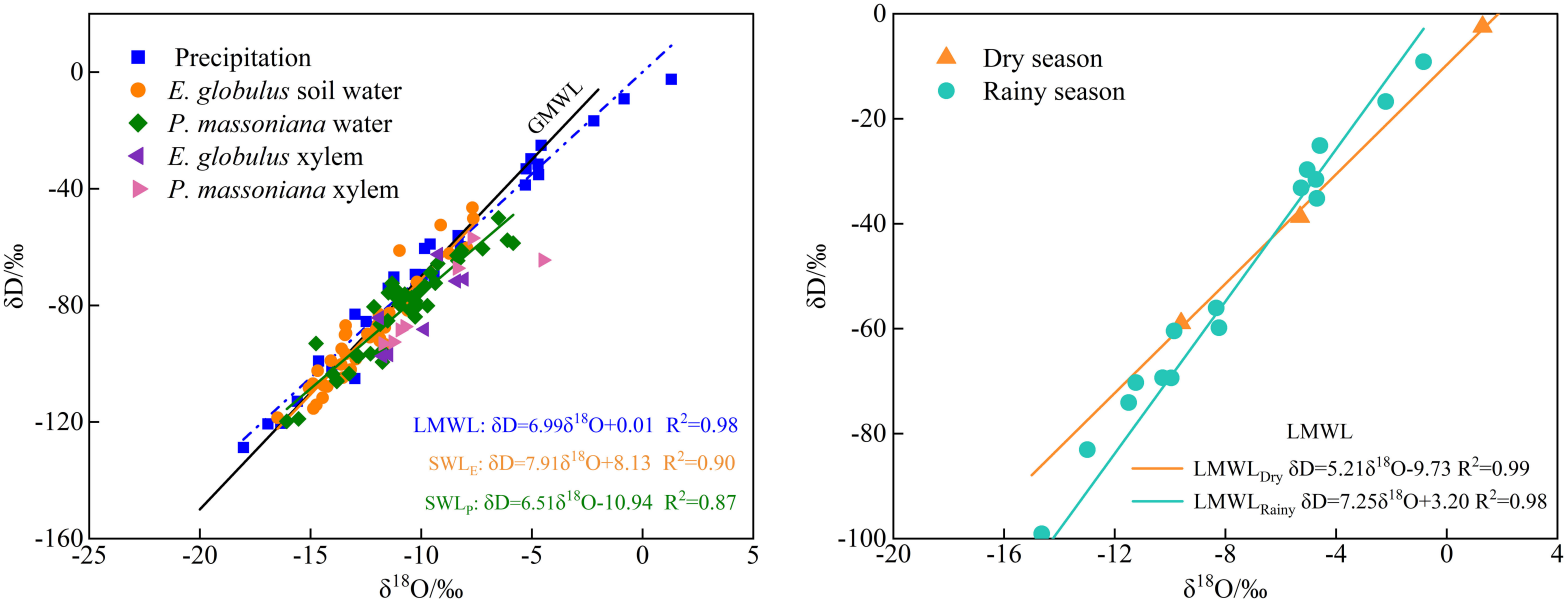
Seasonal patterns of atmospheric precipitation and soil water isotopes in *E. globulus* and *P. massoniana* forests in the study area. The local meteoric water line (LMWL) is derived from the isotopic composition of precipitation in the study area, yielding the equation δD = 6.99δ¹^8^O + 0.01 (R² = 0.98, *p* < 0.01). The soil water line (SWL) is fitted based on the isotopic values of soil water collected from the *E. globulus* and *P. massoniana* plots.

The stable hydrogen and oxygen isotope values of precipitation varied considerably during the measurement period, as shown in **Figure 3**. Precipitation δD and δ¹^8^O values ranged from −128.76‰ to −2.46‰ and from −18.03‰ to 1.29‰, with mean values of −66.19‰ and −9.47‰, respectively. Based on the linear relationship between δD and δ¹^8^O in precipitation samples collected outside the forest, the local meteoric water line (LMWL) for the study area was δD = 6.99 δ¹^8^O + 0.01. The LMWL was δD = 5.21 δ¹^8^O − 9.73 during the dry season and δD = 7.25 δ¹^8^O + 3.20 during the rainy season. Compared with the global meteoric water line, GMWL: δD = 8.0 δ¹^8^O + 10.0, and the Chinese meteoric water line, δD = 7.9 δ¹^8^O + 8.2 (Zheng et al., 1983), both the slope and intercept of the LMWL were lower, indicating evaporative enrichment of precipitation and partial isotopic fractionation during rainfall events in the karst graben basin.

During the observation period, the δD and δ¹^8^O values of soil water in the *E. globulus* and *P. massoniana* stands were distributed around the LMWL. The slope and intercept of the soil water line for the *P. massoniana* stand were lower than those of the LMWL, suggesting that soil water was affected by evaporation enrichment after rainfall infiltration. The δD and δ¹^8^O values of branch water from both species fell within the range of the soil water line, indicating that soil water from different depths was the primary water sources for both species. Comparative analysis of soil water δD and δ¹^8^O values between the two stands showed that the slope of the soil water line was lower in the *P. massoniana* stand than in the *E. globulus* stand, indicating stronger soil evaporative enrichment in the *P. massoniana* stand.

**Figure 4:**
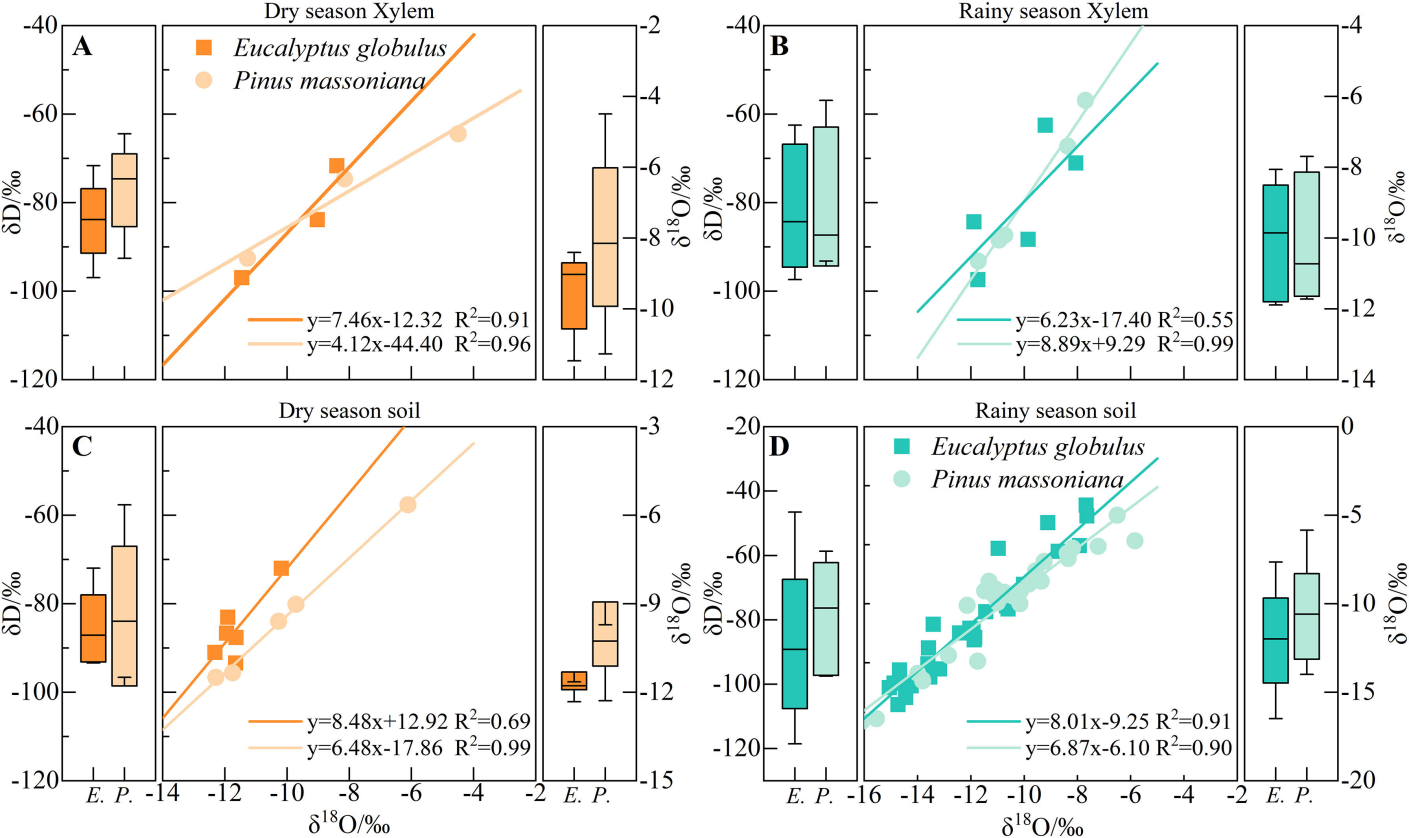
Box plots and scatter plots illustrating the seasonal variations in the isotopic composition of plant xylem water and soil water for *E. globulus* and *P. massoniana* during the dry and rainy seasons. **(A)** Isotopic composition of xylem water during the dry season; **(B)** isotopic composition of xylem water during the rainy season; **(C)** isotopic composition of soil water during the dry season; and **(D)** isotopic composition of soil water during the rainy season. In the box plots, the median is represented by a black line, the box spans the interquartile range, and the whiskers extend to the full range of the data.

The δD and δ¹^8^O values of xylem water differed between seasons for both *E. globulus* and *P. massoniana* (**Figures 4A, B**). During the dry season, mean xylem water δD and δ¹^8^O values were −84.13‰ and −9.63‰, respectively, for *E. globulus*, and −77.21‰ and −7.97‰, respectively, for *P. massoniana*. In the rainy season, the corresponding values were −80.69‰ and −10.15‰ for *E. globulus*, and −78.62‰ and −9.89‰ for *P. massoniana*. In both seasons, mean xylem water δD and δ¹^8^O values were higher in *P. massoniana* than in *E. globulus*. A linear relationship between δD and δ¹^8^O in xylem water was observed for both species in both seasons.

The stable hydrogen and oxygen isotopic composition of soil water also differed seasonally between the two plantation stands (**Figures 4C, D**). During the dry season, mean soil water δD and δ¹^8^O values were −85.59‰ and −11.61‰, respectively, in the *E. globulus* stand, and −82.79‰ and −10.03‰, respectively, in the *P. massoniana* stand. In the rainy season, the corresponding values were −87.49‰ and −12.08‰ in the *E. globulus* stand, and −79.74‰ and −10.71‰ in the *P*. *massoniana* stand. Frequent rainfall events during the rainy season diluted soil water isotopes, resulting in lower mean isotopic values in both stands than in the dry season.

### 3.3 Seasonal variations in water use and transpiration in two typical plantation forest systems

**Figure 5:**
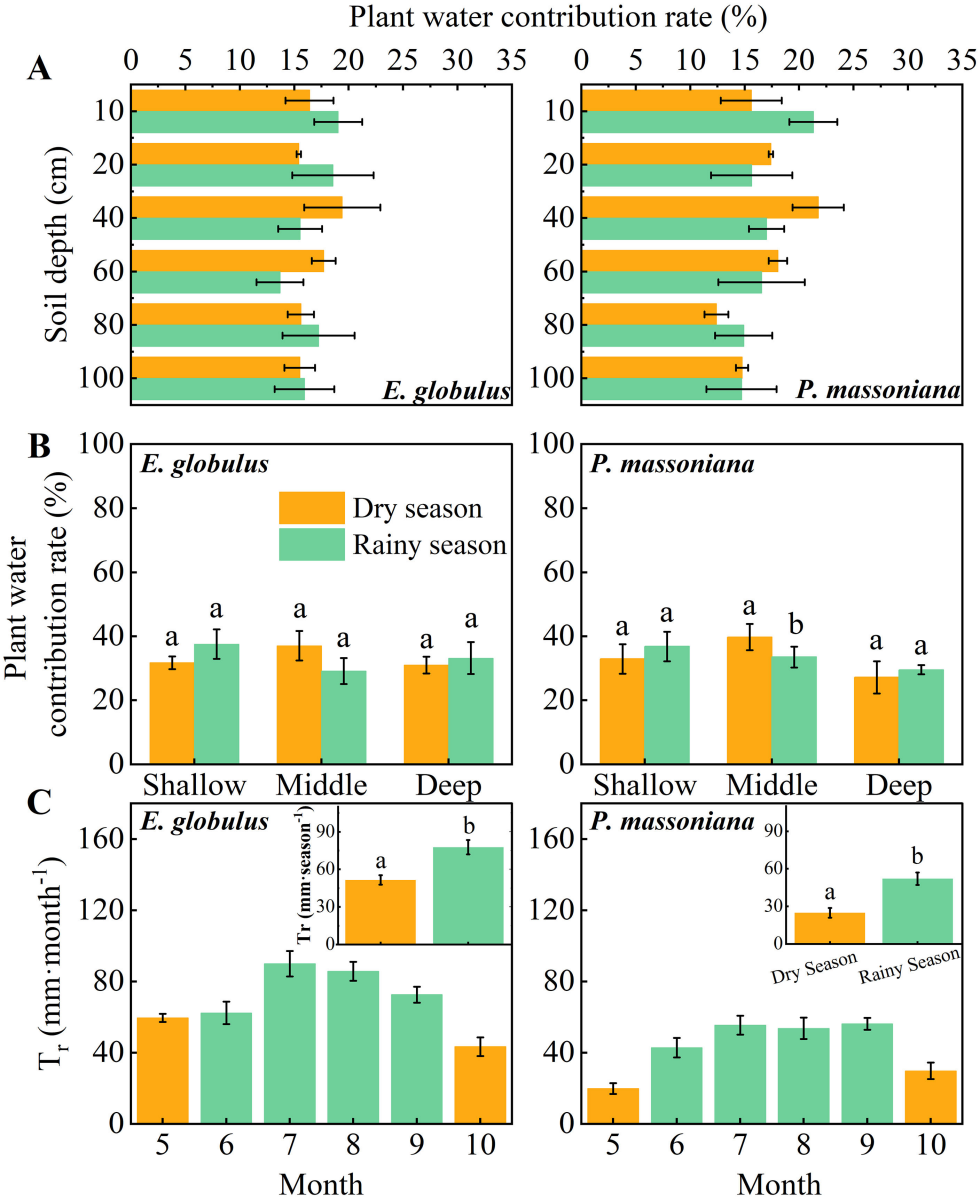
Contributions of soil water from different depths to *E. globulus* and *P. massoniana* and their seasonal variations **(A, B)**. Differences in transpiration between the two species and their seasonal dynamics **(C)**.

Proportional water uptake from different soil layers showed distinct seasonal variations between the *E. globulus* and *P. massoniana* plantations (**Figure 5A**). During the dry season, May and October, *E. globulus* derived most of its water from the middle and deep soil layer, 20–100 cm, which together accounted for 68.2% of total uptake, with the largest contribution from the middle layer 20–60 cm, at 37.1%. In contrast, *P. massoniana* mainly obtained water from the shallow and middle soil layer, 0–60 cm, during the dry season, which together contributed 70.4% of total uptake (**Figure 5B**). As precipitation increased during the rainy season, water uptake patterns shifted in both species. The two plantations showed similar uptake patterns, preferentially absorbing water from the shallow 0–20 cm soil layer, which contributed 37.6% for *E. globulus* and 36.9% for *P. massoniana* (**Figure 5B**). With continuous infiltration of antecedent precipitation, soil water content in deeper layers increased, and both species used water from multiple soil layers.

Transpiration, Tr, also showed clear seasonal patterns in both plantations (**Figure 5C**). Owing to species-specific differences in water use, Tr in the *E. globulus* stand was significantly higher than that in the *P. massoniana* stand, although the magnitude of this difference varied over time. During the observation period, monthly cumulative Tr in the *E. globulus* stand followed an approximately unimodal pattern, with the lowest and highest values recorded in October, 43.42 ± 5.25 mm, and July, 89.96 ± 7.11 mm, respectively. Monthly cumulative Tr in the dry season, 51.50 ± 3.77 mm, was significantly lower than that in the rainy season, 77.67 ± 5.80 mm. For *P. massoniana*, the lowest and highest monthly cumulative Tr values occurred in May, 19.95 ± 3.01 mm, and September, 56.22 ± 3.36 mm, respectively. Total Tr during the observation period was 413.67 mm for the *E. globulus* stand and 258.18 mm for the *P. massoniana* stand, accounting for 60.28% and 37.62% of total precipitation, respectively (*p* < 0.05). Moreover, the most active period of water uptake and Tr for both plantations coincided with the rainy season. Tr differed markedly between the dry and rainy seasons: mean monthly Tr of *E. globulus* was 2.1 times that of *P. massoniana* in the dry season and 1.5 times in the rainy season.

## 4 Discussion

### 4.1 Seasonal variations in isotopes in different water bodies

During the observation period, precipitation δD and δ¹^8^O showed distinct dry-and-rainy-season patterns. Specifically, δD and δ¹^8^O values were relatively high in May and October, corresponding to the dry season, and became more depleted during the rainy season (**Figure 2A**). This pattern suggests that isotopic variability in precipitation was influenced by both air temperature and precipitation amount (Wang et al., 2022; Pei et al., 2023b). Previous studies have also shown that seasonal variation in stable hydrogen and oxygen isotopes in precipitation is common in low-latitude monsoon regions and is mainly driven by seasonal differences in moisture sources, rainfall amount, and evaporative enrichment (Wei and Lin, 1994; Li et al., 2016).

Comparison of δD and δ¹^8^O values among precipitation, xylem water, and soil water revealed distinct isotopic signatures between the dry and rainy seasons. The slope and intercept of the local meteoric water line (LMWL) provide useful information on local climatic and evaporative conditions (Araguás-Araguás et al., 2000; Peng et al., 2004). In this study, both the slope and intercept of the LMWL were lower in the dry season, 5.21 and −9.73, respectively, and in the rainy season, 7.25 and 3.20, respectively, than those of the Chinese meteoric water line, 7.9 and 8.2, and the global meteoric water line, GMWL; 8.0 and 10 (**Figure 3**). These results indicate that precipitation in the karst graben basin experienced stronger evaporative enrichment than is represented by the regional and global meteoric water lines. Further examination of the xylem water line and soil water line for the two plantations showed that during the dry season, both the slope and intercept of the regression line were lower for *P. massoniana* than for *E. globulus* (**Figure 3**). This pattern suggests stronger soil evaporative enrichment in the *P. massoniana* stand than in the *E. globulus* stand.

The isotopic composition of xylem water in *E. globulus* and *P. massoniana* showed clear seasonal variation. During the dry season, *P. massoniana* mainly took up water from the 0–60 cm soil layer (**Figure 5A**). Together with stronger soil evaporation enrichment in the *P. massoniana* stand, this uptake pattern resulted in higher isotopic values in xylem water. Accordingly, xylem water δD and δ¹^8^O values were higher in *P. massoniana* than in *E. globulus* during the dry season (**Figure 4A**). During the rainy season, both species relied mainly on shallow soil water as their primary water source, resulting in more similar xylem water δD and δ¹^8^O values between the two plantations (**Figure 4B**).

**Figure 6:**
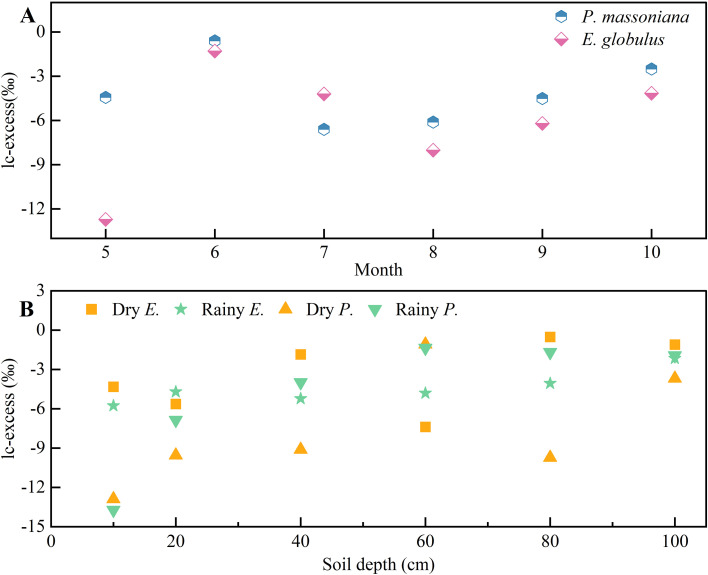
Characteristics of lc-excess in soil water across different layers of the two plantation types. **(A)** Monthly variation in lc-excess of soil water for E. globulus and P. massoniana. **(B)** Variation in lc-excess of soil water with soil depth during the dry and rainy seasons. lc-excess is calculated based on the local meteoric water line.. In the figure, *E.* denotes *E. globulus*, and *P.* denotes *P. massoniana*.

Soil water lc−excess is an important indicator of soil evaporation intensity and can also be used to assess the offset between plant xylem water and local precipitation in δD-δ¹^8^O space (Landwehr and Coplen, 2006; Sprenger et al., 2017). The δD and δ¹^8^O composition of soil water is influenced by precipitation infiltration and soil evaporation, and varies substantially with soil depth and time (Bowen et al., 2018; Pei et al., 2023b). In this study, xylem water δD and δ¹^8^O values in both plantations were consistently below 0‰ (**Figure 6A**). Moreover, lc−excess values in the 0–20 cm soil layer were more negative than those in deeper layers in both stands (**Figure 6B**), supporting the widely observed pattern that surface soils experience stronger evaporative enrichment than deeper soils (Lyu et al., 2021).

### 4.2 Differences in water use strategies and transpiration between the two plantation species

In forest ecosystems, differences in plant species and root distribution depth lead to distinct water use strategies between plantation types, and these strategies often show clear seasonal patterns. In karst graben basins, such biological differences operate within a distinctive ecohydrological context. The dual surface-subsurface hydrological structure, relatively deep soil profile, rapid leakage, limited access to deep groundwater, and precipitation-dependent soil water recharge together create a paradox of deep soils but limited plant-available water. Therefore, the capacity of roots to access water from different soil depths becomes particularly important for sustaining transpiration under seasonal drought.

**Figure 7:**
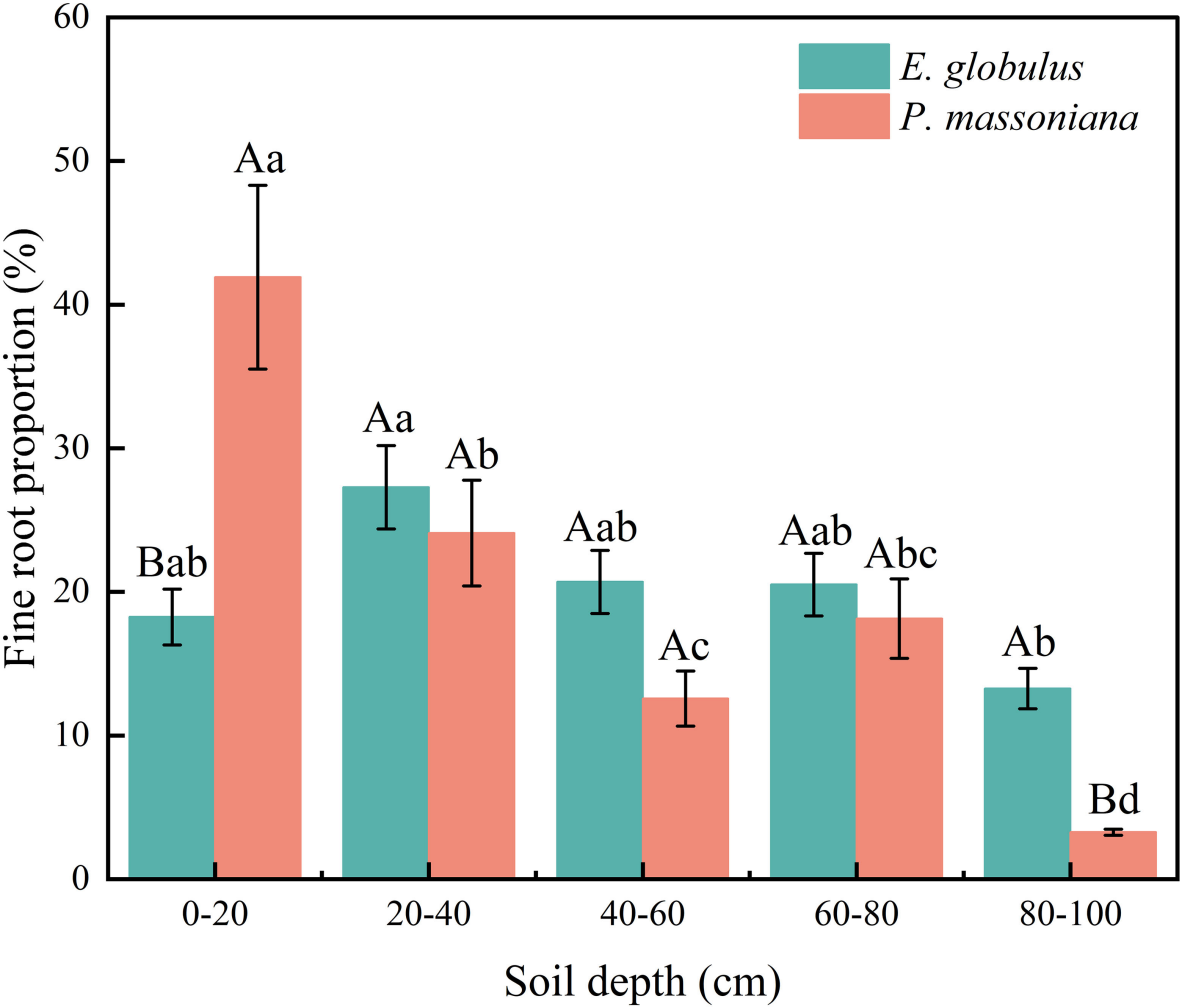
Percentage of fine root biomass at different soil depths in *Eucalyptus globulus* and *Pinus massoniana* plantations. Uppercase letters indicate significant differences between plantations within the same soil layer; lowercase letters indicate significant differences among soil layers within the same plantation (*p* < 0.05).

In this study, during the rainy season when soil water was relatively abundant, both *E. globulus* and *P. massoniana* predominantly used shallow soil water from the 0–20 cm layer. Previous studies have shown that, in karst regions, soil water availability is strongly influenced by precipitation characteristics and root distribution, and that preferential uptake of shallow soil water during the rainy season is often associated with well-developed shallow root systems (Nie et al., 2014). Shallow soil layers are typically richer in organic matter and have a looser structure, allowing plants to acquire water with lower energy expenditure. Thus, when soil water is sufficient, plants tend to use shallow soil water to reduce the energetic cost of water uptake (Cheng et al., 2012). Several factors may explain the differences in water uptake between the two species across the dry and rainy seasons. First, dimorphic root systems play a critical role in seasonal shifts water use (Dai et al., 2023; Liu et al., 2025a; Wen et al., 2025). In this study, the root biomass of *P. massoniana* was concentrated mainly in the 0–40 cm soil layer, accounting for 66.01% of total root biomass, whereas the root system of *E. globulus* was more evenly distributed throughout the 0–100 cm soil profile (**Figure 7**). Consequently, *E. globulus* exhibited a more opportunistic water use strategy in response to seasonal changes in water availability, whereas *P. massoniana* adopted a more conservative strategy by relying mainly on soil water from the 0–60 cm layer to cope with drought stress (Wang et al., 2010; Wu et al., 2021). These findings are consistent with those of Ding et al. (2016), who reported flexible water sources use by *Eucalyptus urophylla × E. grandis* during the dry season in karst regions.

Secondly, precipitation influences plant water use strategies by altering soil water replenishment patterns. During the rainy season, frequent and abundant rainfall maintains high surface soil water availability, allowing plants to readily access water through shallow roots. As the rainy season transitions to the dry season, reduced precipitation leads to progressive soil water depletion and increased drought frequency. Under these conditions of soil water deficit, water uptake by both plantations became constrained, resulting in reduced transpiration (**Figure 5**). This shift also indicates that insufficient precipitation cannot replenish surface soil moisture in a time, forcing roots to extract water from deeper soil layers (Luo et al., 2022; Yang et al., 2025).

Thirdly, species-specific traits, particularly leaf area, also influence water uptake patterns (Wang et al., 2020). *E. globulus* is a broadleaf evergreen species, whereas *P. massoniana* is a needle-leaved conifer. Leaf area is a key determinant of transpiration: a larger leaf area increases the total stomatal surface exposed to the atmosphere and the effective surface for water loss, thereby enhancing transpiration demand (Oren et al., 1999; Bowden and Bauerle, 2008; Li et al., 2009). The contrasting water use strategies of *E. globulus* and *P. massoniana* therefore reflect the coupling of species-specific functional traits with the hydrological structure of the karst graben basin. Differences in canopy architecture, leaf traits, and root distribution may allow the two species to partition soil water sources and buffer seasonal water stress under highly uneven rainfall. Such trait-mediated partitioning is particularly important in karst landscapes, where rapid infiltration, limited shallow soil water retention, and restricted access to deep groundwater can make root distribution a key determinant of seasonal water uptake (Canadell et al., 1996; Nie et al., 2012; Ding et al., 2021). This mechanism provides a plausible explanation for the seasonal adjustment of water uptake by the two plantation species in the study region.

However, our inference was based primarily on soil water within the 0–100 cm profile. Although epikarst water, fissure water, and groundwater can contribute substantially to plant water use in some karst ecosystems (Liu et al., 2014; Nie et al., 2017; Cai et al., 2023), no visible fissures or exposed bedrock were observed in the examined soil profiles, and the groundwater table was deep. Thus, at the scale of this study, soil water was considered the main water source directly accessible to plant roots. Future work should combine isotopic tracing with direct observations of epikarst water, fissure water, groundwater dynamics, and root distribution to determine whether non-soil water sources also contribute to plantation transpiration in karst graben basins.

### 4.3 Implications for water resource management in *E. globulus* and *P. massoniana* plantations

Efficient use of limited water resources remains a central challenge in the ecologically fragile karst regions of Southwest China, particularly under rocky desertification control (Song et al., 2017; Qiu et al., 2023). Afforestation has increased vegetation cover and slowed rocky desertification, but expanding plantation may also intensify soil water consumption in areas with strong seasonal drought and limited soil water storage (Cao, 2011; Chen et al., 2015; Tong et al., 2020; Wu et al., 2021; Cai et al., 2025). Our results showed that both plantations shifted water uptake toward deeper soil layers and reduced transpiration during the dry season, whereas rainy season precipitation promoted shallow soil water use from the 0–20 cm layer and supported higher transpiration. This seasonal shift indicates that precipitation recharge strongly regulates water sources and transpiration in both plantations (Yang et al., 2025). Although *E. globulus* and *P. massoniana* were constrained by low soil water content during the dry season, their distinct water use strategies helped maintain water supply during the observation period.

From a management perspective, afforestation in karst graben basins should be planned according to local soil-water carrying capacity, as determined by soil depth, exposed-rock ratio, seasonal soil water storage, rainfall recharge, and drought intensity (Jiang et al., 2014; Shao et al., 2018). On sites with shallow soils and severe seasonal drought, high-density monocultures of high-water-use species should be avoided unless supported by site-specific water-balance assessments. On basin floors with relatively deep soils and substantial rainy-season recharge, fast-growing species such as *E. globulus* may be considered, but only under controlled planting density and continued monitoring of transpiration demand and soil water depletion. By contrast, species with more conservative water-use strategies, such as *P. massoniana*, may be better suited to sites where shallow to middle soil layers provide the dominant available water source.

Plantation structure should also be diversified. Mixed plantations that include native or locally adapted drought-tolerant species may improve ecosystem stability and reduce the risks associated with single-species water use (Forrester, 2014; Mori et al., 2017; Liu et al., 2025b). Management should further prioritize water retention by protecting litter layers, minimizing soil disturbance, maintaining understory vegetation, and establishing tree-shrub-grass assemblages where appropriate, as the measures can improve surface soil structure, rainfall interception, infiltration, and shallow soil water storage (Wu et al., 2021). Because this study covered only one growing season, the long-term ecological and hydrological consequences of these water use strategies remain uncertain. Future work should combine multi-year monitoring of soil-plant water relations with assessments of water-resource carrying capacity and planting-density experiments across contrasting site conditions. Deep learning approaches could also be used to integrate isotope-based water source information, soil moisture dynamics, and sap flow observations, thereby better characterizing nonlinear plant water-use responses to seasonal water availability (Bandai and Ghezzehei, 2021; Wang et al., 2023). Such efforts will be essential for evaluating the sustainability of plantation ecosystems in karst graben basins and for guiding species selection and spatial planning under a changing climate.

## 5 Conclusion

Understanding the coupling between plant water demand and limited soil water availability is essential for vegetation restoration and rocky desertification control in ecologically fragile karst graben basins. In this study, sap flow monitoring and stable hydrogen and oxygen isotope techniques were used to investigate the water-use mechanisms and transpiration of two typical plantation species, *Eucalyptus globulus* and *Pinus massoniana*, during the growing season in a karst graben basin. *E. globulus* adopted a flexible water use strategy, shifting water uptake toward deeper soil layers, 20–100 cm, and maintaining relatively high transpiration during dry periods, thereby adapting to fluctuating water availability. In contrast, *P. massoniana* exhibited a more conservative water use strategy, absorbing water mainly from shallow to middle soil layers, 0–60 cm, and reducing transpiration under water stress. During the rainy season, both species relied primarily on shallow soil water. Mean monthly transpiration of *E. globulus* was 2.1 and 1.5 times that of *P. massoniana* in the dry and rainy seasons, respectively. The contrasting water-use patterns of the two plantations were associated with differences in root distribution and leaf morphology. Future research should monitor water status and stable isotopes across the soil-plant-atmosphere continuum over longer periods and at multiple spatial scales to better understand how plantation water-use patterns respond to climate variability, thereby providing a stronger theoretical basis for sustainable vegetation restoration in karst graben basins.

## Data Availability

The original contributions presented in the study are included in the article/supplementary material. Further inquiries can be directed to the corresponding author.
